# Reduction of Saltiness and Acrylamide Levels in Palm Sugar-Like Flavouring through Buffer Modification and the Addition of Calcium Chloride

**DOI:** 10.3390/molecules18066792

**Published:** 2013-06-10

**Authors:** Phui Yee Tan, Chin Ping Tan, Faridah Abas, Chun Wai Ho, Wan Aida Wan Mustapha

**Affiliations:** 1 Department of Food Technology, Faculty of Food Science and Technology, Universiti Putra Malaysia, 43400 UPM, Serdang, Selangor, Malaysia; E-Mails: aries_ddj@hotmail.com (P.Y.T.); faridah@food.upm.edu.my (F.A.); 2 Department of Food Science and Nutrition, Faculty of Applied Sciences, UCSI University, No. 1, Jalan Menara Gading, UCSI Heights, Cheras 56000, Kuala Lumpur, Malaysia; E-Mail: cwho@ucsiuniversity.edu.my; 3 School of Chemical Sciences and Food Technology, Faculty of Science and Technology, Universiti Kebangsaan Malaysia, 43600 UKM Bangi, Selangor, Malaysia; E-Mail: wawm@ukm.my

**Keywords:** palm sugar-like flavoring (PSLF), acrylamide, pyrazines, furaneol, high performance liquid chromatography-mass spectrometry (HPLC-MS)

## Abstract

Palm sugar-like flavouring (PSLF) is a type of flavour product that is formed by heating amino acids and sugar under specific heating conditions. Unfortunately, PSLF has a salty taste and contains high amounts of acrylamide. Hence, the objective of this research was to reduce saltiness and acrylamide without negatively affecting the aroma properties of PSLF. A decrease in the sodium phosphate (NaHPO_4_) buffer concentration from 0.20 to 0.02 M was found to reduce sodium to approximately 15% of the level found in original PSLF. A further decrease (~25%) in the sodium content was achieved by removing monobasic sodium phosphate (NaH_2_PO_4_) from the buffer system. Meanwhile, the addition of CaCl_2_ at 20–40 mg/L reduced the acrylamide content in PSLF by as much as 58%. A CaCl_2_ concentration of 20 mg/mL was most favourable as it most efficiently suppressed acrylamide formation while providing an acceptably high flavour yield in PSLF. In view of the high acrylamide content in PSLF, additional work is necessary to further reduce the amount of acrylamide by controlling the asparagine concentration in the precursor mixture.

## 1. Introduction

Palm sugar is famous for its unique flavour and is therefore widely used in many local Asian cuisines and deserts. The traditional production of palm sugar normally involves heating of 50 L of palm sap of the tropical sugar palm tree (*Arenga pinnata*) in a wok pan for a few hours until a concentrate is obtained [1]. Some major limitations of this traditional method include the inconsistent quality of raw palm sap and non-standardised processing methods, which lead to inconsistent quality of the resulting palm sugar. These factors prompted the establishment of a novel approach in which a mixture of amino acids and sugar dissolved in sodium phosphate buffer solution was heated at a specific temperature and time to undergo the Maillard reaction [2]. The resulting final product was named palm sugar-like flavouring (PSLF). Interestingly, PSLF possesses a high amount of flavour compounds and improved aroma stability compared with traditional palm sugar [2]. In addition, PSLF exhibits antioxidant properties. 

However, PSLF possesses a few disadvantages [2]. In terms of taste, PSLF is salty. This saltiness is due to the high concentration (0.20 M) of NaHPO_4_ buffer used in its production process. The NaHPO_4_ buffer system consists of two buffer salts, monobasic (NaH_2_PO_4_) and dibasic (Na_2_HPO_4_) sodium phosphate. These two buffer salts were used to adjust the pH of the solution in which the precursors were dissolved. The sodium content of PSLF could be reduced by modifying the preparation of the sodium phosphate buffer solution by reducing the buffer concentration and removing one of the buffer salts (NaH_2_PO_4_). 

Moreover, in terms of safety, PSLF was found to contain significant amount of acrylamide. Acrylamide is a genotoxic and neurotoxic compound that has been classified as a probable human carcinogen [3]. It is commonly present in various heat-processed foods, namely potato crisps, breads and cookies. The formation of acrylamide is primarily related to the Maillard reaction between amino acids, primarily asparagine, and a carbonyl group of a reducing sugar at high temperature [4]. Process temperature, time, pH and precursor (reducing sugar and asparagine) concentration are among the common factors affecting acrylamide formation in foods [5,6]. Numerous research studies [7,8] have aimed to manipulate these factors to minimise acrylamide formation in different foods. In addition, monovalent (Na^+^) and divalent metal salts (Ca^2+^ and Mg^2+^) have been added to different food systems to inhibit and reduce acrylamide formation. Divalent metal salts such as calcium chloride (CaCl_2_) and magnesium chloride (MgCl_2_) effectively inhibit acrylamide formation [8,9,10]. Thus, calcium chloride was selected in the present study for addition to the PSLF precursor to reduce acrylamide formation.

In this study, an experiment was conducted by modifying the buffer solution (reduction of the buffer concentration and removal of NaH_2_PO_4_), followed by the addition of different concentrations of calcium chloride to the PSLF precursor mixture in an attempt to reduce sodium and acrylamide without negatively affecting the aroma profile of PSLF. Hence, the main objective of this study was to investigate the effect of buffer modification and the addition of different concentrations of CaCl_2_ on saltiness, acrylamide and the flavour profile of PSLF.

## 2. Results and Discussion

### 2.1. Effect of NaHPO_4_ Buffer (Na_2_HPO_4_/NaH_2_PO_4_) Modification

As presented in Table 1, reducing the Na_2_HPO_4_/NaH_2_PO_4_ concentration from 0.20 M to 0.02 M at a fixed asparagine concentration significantly (*p* < 0.05) reduced the sodium content (15%). At 0.02 M Na_2_HPO_4_, removal of NaH_2_PO_4_ from the buffer system further decreased sodium content up to approximately 25% compared to 0.02 M Na_2_HPO_4_/NaH_2_PO_4_. In addition, modification of the buffer system significantly (*p* < 0.05) decreased the initial pH of the precursor mixture. However, in contrast to previous studies [10,11], a decrease in the initial pH did not contribute to acrylamide reduction. In fact, acrylamide content significantly (*p* < 0.05) increased, up to approximately 48%, with the reduced buffer concentration. Moreover, a two-fold increase in acrylamide was observed upon removal of monobasic NaH_2_PO_4_. In this scenario, pH reduction did not play as important a role as the NaHPO_4_ buffer composition in reducing acrylamide formation in PSLF. Na^+^ salt has been reported to reduce acrylamide formation [12]; thus, a decrease in the Na_2_HPO_4_/NaH_2_PO_4_ concentration and removal of monobasic NaH_2_PO_4_ would decrease the concentration of Na^+^ in the mixture, which would consequently reduce the inhibition on acrylamide formation in PSLF.

**Table 1:** Effect of sodium phosphate buffer modification.

	Palm Sugar-Like Flavourings		
	0.20 M Na_2_HPO_4_/NaH_2_PO_4_ (Original)	0.02 M Na_2_HPO_4_/NaH_2_PO_4_	0.02 M Na_2_HPO_4_
Sodium Content (mg/g FW)	72.82 ± 2.67 ^A^	61.51 ± 4.08 ^B^	45.90 ± 0.55 ^C^
Initial pH	7.50 ± 0.02 ^A^	7.01 ± 0.01 ^B^	6.34 ± 0.04 ^C^
Acrylamide Content (ppm)	40.35 ± 2.85 ^C^	59.64 ± 5.24 ^B^	191.38 ± 22.21 ^A^
Flavour Components (ppb)			
EDMP	393.14 ± 18.17 ^A^	77.41 ± 8.15 ^B^	ND
DEMP	52.27 ± 5.46 ^C^	164.34 ± 7.03 ^A^	122.66 ± 7.93 ^B^
5*H*-5-Methyl-6, 7-dihydroxycyclopenta[b] pyrazine	1262.94 ± 141.77 ^A^	1126.93 ± 119.25 ^A^	1119.99 ± 69.22 ^B^
Furaneol	39.75 ± 4.52 ^A^	51.67 ± 5.22 ^A^	160.71 ± 14.19 ^B^

Na_2_HPO_4_/NaH_2_PO_4_: sodium phosphate buffer system; EDMP: 2-ethyl-3,5-dimethylpyrazine; DEMP: 2,3-diethyl-5-methylpyrazine; ND: not detected; ^A−C^ Different letters indicate significant difference (*p* < 0.05) of results.

In terms of flavour, the overall flavour profile of PSLF prepared with 0.02 M NaHPO_4_ was higher than that of PSLF prepared with 0.20 M Na_2_HPO_4_/NaH_2_PO_4_. This might be due to the significant (*p* < 0.05) reduction of the initial pH, as pH is a critical factor affecting the Maillard reaction pathway, thus influencing the production of flavour compounds [13]. Removal of NaH_2_PO_4_ from the buffer system adversely affected pyrazine components compared to PSLF using 0.02 M Na_2_HPO_4_/NaH_2_PO_4_, with the loss of one pyrazine component (EDMP) despite the further reduction of initial pH. In general, buffer could act as a catalyst for the Maillard reaction [14]. Thus, the absence of one of the buffer components (NaH_2_PO_4_) would therefore influence the Maillard reaction-mediated formation of flavour compounds. Because the main priority of the buffer modification stage was to reduce the PSLF sodium content, 0.02 M Na_2_HPO_4_, which had the lowest sodium content, was selected for use in subsequent experiments to further investigate the effect of CaCl_2_. 

### 2.2. Effect of CaCl_2_

The addition of CaCl_2_ at various levels (0–80 mg/L) did not cause any significant (*p* > 0.05) change in the initial pH of the PSLF precursor mixture. Consequently, the impact of initial pH on all the responses was negligible.

#### 2.2.1. Effect of CaCl_2_ on Acrylamide Formation in PSLF

**Figure 1:**
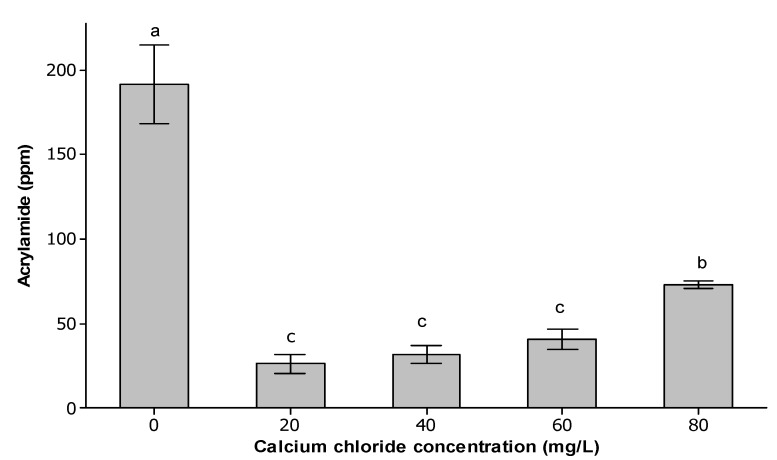
Acrylamide content in PSLF of different CaCl_2_ concentrations.

In comparison with PSLF without CaCl_2_, the addition of 20 mg/L CaCl_2_ significantly (*p* < 0.05) suppressed acrylamide formation in PSLF by nine-fold (Figure 1). Based on previous research studies [4,15,16], acrylamide has been proposed to primarily originate from the Schiff’s base of N-glycosyl-asparagine, which is formed via Maillard reaction between asparagine and a carbonyl group of a reducing sugar. In this context, Gokmen and Senyuva [9] revealed that the addition of equimolar Ca^2+^ in an equimolar asparagine-fructose model system successfully removed the Schiff’s base and thus acrylamide after heating at 150 °C for 15 min. With this regard, the reduction of acrylamide in PSLF might be attributable to the interaction between Ca^2+^ and asparagine, which results in a decrease in the amount of asparagine available to form N-glycosylasparagine in the reaction mixture. 

Surprisingly, acrylamide levels were found to increase progressively as the CaCl_2_ concentration increased from 20 to 80 mg/L CaCl_2_. No significant (*p* > 0.05) difference in acrylamide content was observed for 20–60 mg/L CaCl_2_. This result is in complete contrast to earlier studies [8,10,17] in which increases in the CaCl_2_ concentration inhibited acrylamide formation. However, according to Casado *et al.* [18], the addition of 50 mM CaCl_2_ increased acrylamide yield in olives by up to 24%, and it was suggested that formation of acrylamide in olives might follow a different pathway. In this aspect, it is possible that when the concentration of CaCl_2_ increased to 80 mg/L, the interaction between Ca^2+^ and the other reactants occurred differently, which somehow favoured increased production of Maillard reaction intermediates, particularly N-glycosylasparagine [9], thus enhancing acrylamide formation in PSLF.

In general, the overall acrylamide content in PSLFs with 20–80 mg/L CaCl_2_ was still extremely high, with a minimum concentration of up to a part per million (ppm) at 20 mg/L CaCl_2_. The revealed amount of acrylamide in PSLF was even higher than that of other acrylamide-containing foods, for instance, potato crisps and French fries [6,10]. In this scenario, the presence of asparagine as one of the PSLF precursors was suspected to be the prior factor leading to this high concentration of acrylamide in PSLF. Thus, reduction of acrylamide could be done by reducing or eliminating the asparagine in the PSLF precursor mixture. We plan to study the effect of elimination or reduction of asparagine concentration and the interaction effect between asparagine and CaCl_2_ concentrations in the future using response surface methodology (RSM). 

#### 2.2.2. Effect of CaCl_2_ on Flavour Profile in PSLF

**Figure 2:**
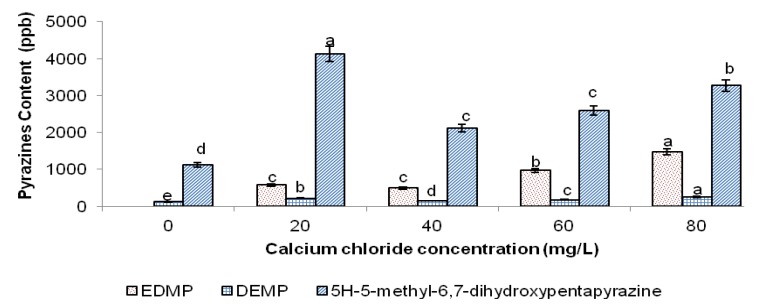
Concentration of pyrazines (EDMP, DEMP and 5*H*-5-methyl-6,7-dihydroxypentapyrazine) in PSLF of different CaCl_2_ concentrations.

**Figure 3:**
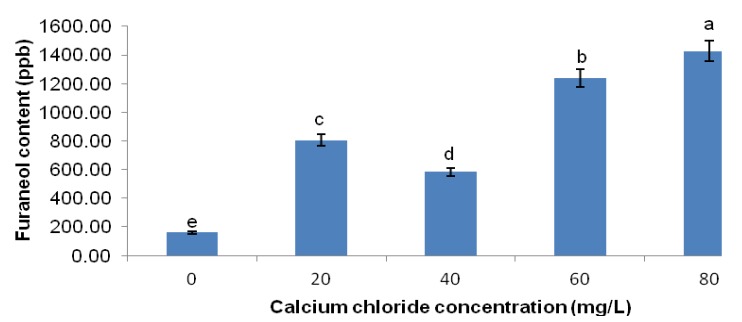
Furaneol content in PSLF of different CaCl_2_ concentrations.

Interestingly, the addition of CaCl_2_ (20–80 mg/L) improved the overall flavour quality of PSLF compared to PSLF without CaCl_2_ (Figures 2 and Figures 3). The addition of 20 mg/L CaCl_2_ induced an up to five-fold increase in pyrazine content and a nine-fold increase in furaneol content in PSLF. However, the pyrazine and furaneol content decreased substantially at 40 mg/L CaCl_2_ and increased as the CaCl_2_ concentration increased beyond 40 mg/L. Overall, 80 mg/L CaCl_2_ provided the best flavour quality with the highest overall flavour concentration in PSLF. 

In general, the Maillard reaction has been the prevalent route for the generation of a wide range of flavour compounds. N-heterocyclics, namely EDMP and DEMP, were reported to be responsible for the odour of cheese products as well as roasted coffee and beef [19]. In addition, 5*H*-5-methyl-6,7-dihydrocyclopentapyrazine is the impact aroma compound in roasted nuts and cocoa, while furaneol is imperative for the sweet caramel-like flavour in processed foods such as soy sauce [20]. All of these flavour compounds were reported to be the potent aroma components that contribute to the unique odour of PSLF [1].

The acceptably high flavour content of PSLF at 20 mg/L CaCl_2_ implied that Ca^2+^ at a concentration of 20 mg/L somehow tends to promote the formation of pyrazines and furaneol through the Maillard reaction in PSLF. Given that the Maillard reaction involves an abundance of complex reaction pathways, the availability of different precursors would control which route predominates [21]. In the present study, asparagine was the major amino acid component in the PSLF precursor mixture. Hence, its availability in the PSLF precursor mixture was believed to be of great impact on the course of the Maillard reaction. As in the formation of acrylamide, the Schiff’s base derived from asparagine is a key intermediate in the generation of pyrazines [22]. In addition, asparagine has been reported to yield more pyrazines than other amino acid compounds in an amino acid-glucose model system [23]. The presence of 20 mg/L CaCl_2_ might favour the formation of volatile compounds from asparagine to predominate instead of the formation of acrylamide. This may be one of the factors contributing to the suppression of acrylamide content in PSLF in the presence of 20 mg/L CaCl_2_. 

The sudden decrease in the overall flavour yield of PSLF with 40 mg/L CaCl_2_ might be due to the increased Ca^2+^ leading to further interference with the Maillard reaction in the asparagine-mediated Maillard reaction pathway. The increase in both pyrazines and furaneol in PSLF at 40-80 mg/L CaCl_2_ further supports the promotion of pyrazine and furaneol formation by the CaCl_2_-mediated increase in the yield of Maillard reaction intermediates. 

#### 2.2.3. Effect of CaCl_2_ on Sodium Content in PSLF

**Figure 4:**
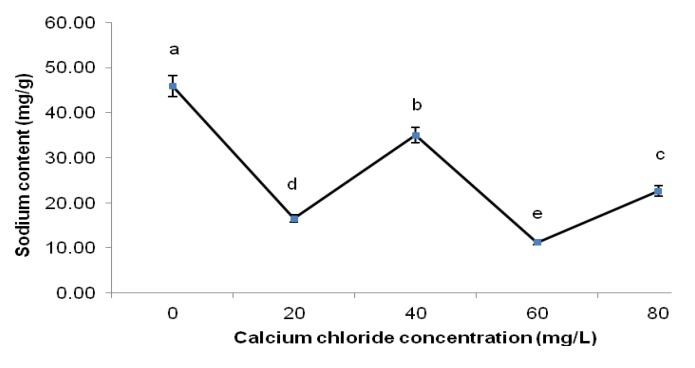
Sodium content of PSLF of different calcium chloride concentrations.

With increasing CaCl_2_ concentration, the PSLF sodium content displayed a fluctuating trend. In general, the addition of CaCl_2_ to the precursor mixture reduced the sodium content in PSLF compared to PSLF without CaCl_2_, as depicted in Figure 4. In particular, 20 and 60 mg/L CaCl_2_ were most efficient in suppressing sodium content in PSLF by approximately 65% and 76%, respectively. This finding indicates the desirable efficiency of CaCl_2_ in suppressing sodium content in PSLF. However, the actual mechanism by which CaCl_2_ reduces sodium content is unknown. 

## 3. Experimental

### 3.1. Materials

L-Arginine, L-asparagine, L-glutamine, L-lysine, sucrose, disodium hydrogen phosphate heptahydrate (Na_2_HPO_4_·7H_2_O), sodium dihydrogen phosphate dihydrate (NaH_2_PO_4_·2H_2_O), and calcium chloride dihydrate (CaCl_2_·2H_2_O) were supplied by Merck (Darmstadt, Germany). All precursor chemicals were of food and pharmacopoeia grade. Acrylamide (≥ 99.9%) and ^13^C_3_-acrylamide (isotopic purity 99%) were purchased from Merck and Cambridge Isotope Laboratories (Andover, MA, USA), respectively. The following flavour compound reference standards for gas chromatography, with a purity of 97–99%, were obtained from Sigma-Aldrich Chemie GmbH (Steinheim, Germany): 2-ethyl-3,5-dimethylpyrazine (EDMP), 2,3-diethyl-5-methylpyrazine (DEMP), 5*H*-5-methyl-6,7-dihydrocyclopenta[b]pyrazine and 4-hydroxy-2,5-dimethyl-3(2*H*)-furanone (furaneol). HPLC grade methanol, analytical grade concentrated hydrochloric acid and formic acid (98%) were purchased from Fisher Scientific (Loughborough, UK). Silicone oil for the oil bath was supplied by Merck. Bond Elut AccuCAT (200 mg, 3 mL) SPE columns were purchased from Varian (Varian Medical Systems, Palo Alto, CA, USA). Ultrapure water (Elga, Buckinghamshire, UK) was used for acrylamide analysis, while deionised water (Sartorius AG, Göttingen, Germany) was used throughout the whole experiment. 

### 3.2. Preparation of PSLF

#### 3.2.1. Original Formula (Na_2_HPO_4_/NaH_2_PO_4_)

The method of preparation of original PSLF was adopted from Ho [2]; a NaHPO_4_ buffer solution of pH 7.86 was first prepared from stock solutions of 0.2 M dibasic (Na_2_HPO_4_) and 0.2 M monobasic (NaH_2_PO_4_) sodium phosphate solutions. Sucrose, l-glutamine, l-asparagine, l-arginine and L-lysine of various molar concentrations were subsequently dissolved in the Na_2_HPO_4_/NaH_2_PO_4_ buffer solution. The mixture solution was transferred to a 500 mL reaction vessel and heated in a thermostated oil bath (Model XMTD-701, Nuohai, Chongqing, China) at 143 °C for 116 min. Heating of the mixture was homogenised with an overhead stirrer (IKA, Staufen, Germany) at a speed of 500 rpm. At the end of heating, a concentrate formed that crystallised and formed a solid upon cooling. 

#### 3.2.2. Buffer Modification

##### 3.2.2.1. Reduced Buffer Concentration (0.02 M Na_2_HPO_4_/NaH_2_PO_4_)

All preparation methods were the same as that of the original method, with the exception that the concentration of the sodium phosphate stock solutions (Na_2_HPO_4_ and NaH_2_PO_4_) was reduced from 0.20 M to 0.02 M.

##### 3.2.2.2. Removal of NaH_2_PO_4_ (0.02 M Na_2_HPO_4_)

In the attempt to further reduce sodium content, monobasic sodium phosphate (NaH_2_PO_4_) was removed from the buffer system. Instead of using both mono- and dibasic, only 0.02 M dibasic sodium phosphate (Na_2_HPO_4_) stock solution was used to prepare a buffer solution of pH 7.86. Precursor concentrations, heating conditions and procedures were fixed as Section 2.2.1.

##### 3.2.2.3. Addition of CaCl_2_

Buffer solution (pH 7.86) was prepared from a stock solution of 0.02 M Na_2_HPO_4_. Precursors and various concentrations of CaCl_2_ (20, 40, 60 and 80 mg/L) were added to the solution, which was subjected to the same heating conditions of Section 2.2.1. PSLF without CaCl_2_ with 0.02 M Na_2_HPO_4_ was treated as a negative control for this stage. The initial pH of the precursor mixtures was measured (PT-10, Sartorius AG) prior to heating. All PSLF samples were ground at 3 mm mesh size, kept in glass bottles tightly sealed with parafilm and stored at −20 °C until further analysis.

### 3.3. Analyses

#### 3.3.1. Acrylamide Analysis

##### 3.3.1.1. Extraction

The acrylamide analysis method was adapted from Serpen and Gokmen [24] with slight modifications. A quantity of finely ground PSLF (1 g) was spiked with 1 mg/mL ^13^C_3_-acrylamide (100 µL) in a 50 mL polypropylene centrifuge tube. A volume of water (9 mL) was added to the sample, and extraction was performed with a vortex mixer for 3 min. The aliquot was then microcentrifuged at 13, 400 × *g* (Eppendorf Ag Minispin Centrifuge, Hamburg, Germany) for 10 min (25 °C). Solid phase extraction (SPE) clean-up was subsequently conducted. Two millilitres of the extract was loaded onto a Bond Elut-AccuCAT SPE cartridge that was preconditioned with 3 mL of methanol and 3 mL of water. The loaded extract was discarded. Two millilitres of water was loaded and eluted drop by drop. The first few drops of eluate were discarded while the remaining eluate was collected and filtered through a 0.22 µm nylon filter. The filtrate was then subjected to HPLC-MS analysis. PSLF was spiked with 50, 200 and 800 ng/mL of a 1 mg/mL acrylamide stock solution and subjected to extraction for a recovery test. 

##### 3.3.1.2. HPLC-MS Analysis

Acrylamide was analysed with a Thermo Scientific Quantum Ultra HPLC system (Thermo Fisher Scientific Co., Ltd, Waltham, MA, USA) coupled to an atmospheric pressure chemical ionisation (APCI) triple quadrupole mass spectrometer. Separation was performed on an Atlantis T3 column (150 mm × 4.6 mm, 3 µm, Waters, Milford, MA, USA) with an isocratic elution with a mobile phase of 0.1% formic acid in water. The flow rate was fixed at 0.3 mL/min (25 °C). Selected reaction monitoring (SRM) was chosen in positive ion scanning mode with the following analysis parameters: discharge current of 4 A; sheath gas pressure of 30 a.u.; ion sweep gas pressure of 1 a.u.; auxiliary gas pressure of 12 a.u.; and vaporiser and capillary temperatures of 375 °C and 250 °C, respectively. The limit of detection (LOD) at a signal/noise ratio of 1:3 was 2 ppb, while the limit of quantitation (LOQ) at a signal/noise ratio of 1:10 was 10 ppb. The stability of the system was verified before each analysis by injecting a standard.

##### 3.3.1.3. Quantification

Working standard solutions of acrylamide (5-1,000 ng/mL) were prepared by serial dilution of a stock solution (1 mg/mL) with ultrapure water. Each standard was spiked with 100 ng/mL of ^13^C_3_-acrylamide as an internal standard and was kept at 4 °C before use. Quantification of acrylamide was based on the peak area ratio of ion transitions of m/s 72 > 55 (acrylamide) and m/s 75 > 58 (^13^C_3_-acrylamide). The established calibration curve had good linearity, with *R^2^* > 0.99.

#### 3.3.2. Flavour Analysis

##### 3.3.2.1. Extraction of Flavour Compounds

The method of flavour analysis was based on Ho *et al.* [1] by employing solid phase micro-extraction (SPME) to extract the flavour components of PSLFs. A 50/30 µm divinylbenzene/ carboxen/polydimethylsiloxane SPME fibre (Supelco, Bellefonte, PA, USA) was selected to provide optimal efficiency in extracting the main flavour compounds of PSLFs (pyrazines and furans). A 1 g quantity of ground PSLF was weighed into an SPME vial equilibrated in a water bath at 50 °C for 10 min. The SPME fibre was inserted into the vial and exposed to the sample for another 10 min by means of a manual SPME holder. The fibre was then injected manually into the injector port of the gas chromatograph for flavour analysis. 

##### 3.3.2.2. Gas-Chromatography-Flame Ionisation Detector (GC-FID) Analysis

Flavour analysis was performed by using an Agilent 7890A GC-FID system (Agilent Technologies, Santa Clara, CA, USA). The SPME fibre was conditioned at 250 °C for 30 min before the first sample analysis. After injecting and exposing the fibre into the injector port, flavour compounds were thermally desorbed in splitless mode through an SPME inlet liner (0.75 mm i.d., Supelco). The fibre was kept in the SPME holder after exposure for 5 min in the injector port. Analytical separation was performed with a non-polar HP-5MS capillary column (30 m, 0.25 mm i.d., 0.25 µm film thickness) purchased from J&W Scientific, Agilent Technologies. Hydrogen gas was used as the carrier gas at a constant flow rate (2 mL/min). The respective injector and detector temperatures were 240 °C and 280 °C. The column temperature was programmed to proceed from an initial temperature of 50 °C (held for 2 min) to 80 °C (held for 1 min) at a rate of 20 °C/min. The temperature was then increased to 100 °C (held for 1 min) at 20 °C/min and finally to 230 °C (held for 2 min) at 30 °C/min. 

##### 3.3.2.3. Identification and Quantification

Flavour compounds (EDMP, DEMP, 5*H*-5-methyl-6,7-dihydrocyclopenta[b]pyrazine and furaneol) were identified according to the retention time of standards. Working standard solutions were prepared by serial dilution of 1,000 ppm stock solutions. The concentration of the compounds was calculated from the respective external calibration curves. 

#### 3.3.3. Sodium Analysis

##### 3.3.3.1. Dry Ashing

The analysis method for sodium was adapted from AACC [25], with minor modifications. A 0.5 g quantity of ground PSLF was weighed into a crucible and placed into a furnace, which was gradually heated to 550 °C. The temperature was maintained for approximately 5 h until a white or grey residue was formed. The ashed residue was cooled in a desiccator and dissolved in 10 mL 3 N HCl. The solution was subsequently heated on a hot plate until the release of gas ceased. Finally, the solution was diluted into a 100 mL volumetric flask with ultrapure water and subjected to analysis by flame atomic absorption spectrometry (FAAS).

##### 3.3.3.2. FAAS Analysis

FAAS was performed on a Thermo Scientific atomic absorption spectrometer (Waltham, MA, USA). A sodium hollow cathode lamp was used at a wavelength of 589.0 nm. An external calibration curve was constructed. Sample solutions were diluted appropriately so that the absorbance was within the linear range of the calibration curve. Sodium concentration in PSLF was calculated on wet weight basis in mg/g fresh weight (FW) unit. 

#### 3.3.4. Statistical Analysis

All experiments and/or analyses were conducted in triplicate. The data were represented as the means ± standard deviation and were analysed via One-way analysis of variance (ANOVA) using Minitab Software (released 14.1; Minitab Inc., State College, PA, USA). Significant differences were determined at *p* < 0.05. 

## 4. Conclusions

Briefly, buffer modification experiments demonstrated a significant (*p* < 0.05) reduction of sodium content compared with original PSLF. The removal of 0.02 M NaH_2_PO_4_ from the phosphate buffer system diminished the overall flavour yield of PSLF, but the aroma profile of PSLF was enhanced by the addition of CaCl_2_. The absence of CaCl_2_ resulted in a high acrylamide content and a reduced number of flavour compounds. A CaCl_2_ concentration of 20 mg/L appeared to be most efficient in reducing acrylamide, with a nine-fold decrease, while providing considerably high aroma content based on the concentrations of EDMP (577.15 ± 50.01 ppb), DEMP (219.01 ± 8.34 ppb), 5*H*-5-methyl-6,7-dihydrocyclopenta[b]pyrazine (4,137.17 ± 145.82 ppb) and furaneol (804.36 ± 6.90 ppb). In addition, 80 mg/L CaCl_2_ yielded the best flavour quality in PSLF but induced the formation of a high amount of acrylamide. With respect to the overall high amount of acrylamide in PSLFs with 20–80 mg/L CaCl_2_, further study can be conducted via manipulation of asparagine concentration to further reduce the level of acrylamide in PSLF. For our future work, the effect of asparagine concentration and its interaction with CaCl_2_ concentration will be investigated via RSM. In the sensory aspect, it is anticipated that the addition of CaCl_2_ and reduction of saltiness will lead to a bitter aftertaste or some undesirable taste of the product. Hence, sensory tests will also be carried out after achieving the minimum level of acrylamide in PSLF.
